# Reconstructing the phylogeny of Blattodea: robust support for interfamilial relationships and major clades

**DOI:** 10.1038/s41598-017-04243-1

**Published:** 2017-06-20

**Authors:** Zongqing Wang, Yan Shi, Zhiwei Qiu, Yanli Che, Nathan Lo

**Affiliations:** 1 College of Plant Protection, Southwest University, Beibei, Chongqing China; 2 School of Life and Environmental Sciences, University of Sydney, Sydney, New South Wales Australia

## Abstract

Cockroaches are among the most recognizable of all insects. In addition to their role as pests, they play a key ecological role as decomposers. Despite numerous studies of cockroach phylogeny in recent decades, relationships among most major lineages are yet to be resolved. Here we examine phylogenetic relationships among cockroaches based on five genes (mitochondrial 12S rRNA, 16S rRNA, COII; nuclear 28S rRNA and histone H3), and infer divergence times on the basis of 8 fossils. We included in our analyses sequences from 52 new species collected in China, representing 7 families. These were combined with data from a recent study that examined these same genes from 49 species, resulting in a significant increase in taxa analysed. Three major lineages, Corydioidea, Blaberoidea, and Blattoidea were recovered, the latter comprising Blattidae, Tryonicidae, Lamproblattidae, Anaplectidae, Cryptocercidae and Isoptera. The estimated age of the split between Mantodea and Blattodea ranged from 204.3 Ma to 289.1 Ma. Corydioidea was estimated to have diverged 209.7 Ma (180.5–244.3 Ma 95% confidence interval [CI]) from the remaining Blattodea. The clade Blattoidea diverged from their sister group, Blaberoidea, around 198.3 Ma (173.1–229.1 Ma). The addition of the extra taxa in this study has resulted in significantly higher levels of support for a number of previously recognized groupings.

## Introduction

Cockroaches are considered to play a key role in terrestrial ecosystems, recycling dead plants, dead animals and excrement and contributing to ecosystem functioning via the breakdown of organic matter and the release of nutrients^1^. The morphologically and ecologically diverse group Blattodea including Isoptera is widely accepted to be a monophyletic^2–13^.

In recent decades a number of studies have examined the phylogeny of Blattodea based on morphological characters^6,14–16^, molecular data^3,7–9,11,13,17–19^, or both^10,12^. Taken together, these studies displayed some consistent relationships, including Ectobiidae (=Blattellidae) being paraphyletic with respect to Blaberidae^6,7,10–13,19^, and Isoptera being placed within Blattodea as sister to Cryptocercidae (morphological methods^6^; molecular methods^3,7,8,11,12,17,19^; combined data^10–12^). The monophyly of termites and their closest relatives *Cryptocercus* is supported by strong synapomorphies, such as xylophagy, biparental care, proctodeal trophallaxis and a rich and highly specific hindgut fauna of flagellates^20–22^.

Despite these advances, the evolutionary relationships among the main lineages of Blattodea have yet to be well resolved, and a number of other results from previous studies remain under discussion. These include: (i) the proposal that Tryonicidae and Lamproblattidae are given family status and excluded from Blattidae^6^; (ii) the proposed sister grouping between Nocticolidae and Corydiidae (=Polyphagidae)^11^; (iii) the sister group relationships between Lamproblattidae and Blattidae^12^; (iv) the sister group of Cryptocercidae + Isoptera, which may be either Tryonicidae, *Anaplecta*, or Tryonicidae + *Anaplecta* ^12^.

Although the Nocticolidae are generally accepted to be a monophyletic group, the placement of Nocticolidae and the relationships with Corydiidae have been debated over the last 20 years. Grandcolas^15^ proposed that Nocticolidae should be lowered to the subfamily level and be synonymised with Latindiinae. In most other studies, Nocticolidae were recovered as the sister group to Corydiidae^7,9,11,19^. When additional Latindiinae taxa were included, Nocticolidae was recovered to be the sister group to *Latindia* + *Paralatindia* ^12,13^.

In this study, we sequenced three mitochondrial **(**12S rRNA, 16S rRNA and COII) genes and two nuclear **(**28S rRNA and Histone H3) genes from 52 blattarian (mainly Ectobiidae, Blaberidae and Blattidae) species collected in China, including representatives of three important genera: *Anaplecta*, *Nocticola* and *Cryptocercus*. Combining these sequences with previously published sequences, and using 8 fossils, we performed phylogenetic and divergence date analyses, and inferred the biogeographic history and timescale of evolution within Blattodea.

## Material and Methods

### DNA extraction, amplification, purification and sequencing

We sampled 5 genes of 52 species (Table S1) from Blattodea in this study: mitochondrial 12S rRNA, 16S rRNA, COII, nuclear 28S rRNA and Histone H3. Total DNA was extracted from hindleg tissues of samples preserved in 100% ethanol. The extraction procedure was according to the TIANamp Genomic DNA Kit (Tiangen Biotech, Beijing). Fragments of 12S rRNA, 16S rRNA, COII, 28S rRNA and H3 were amplified using PCR. Primers for the amplifications of these partial genes are given in Table 1.

**Table 1:** Primers used to generate sequences.

Genes	Forward/ Reverse	Primer name	Sequence(5′-3′)	Reference
12S	F	12S forward	ATCTATGTTACGACTTAT	Inward *et al*.^7^
	R	12S reverse	AAACTAGGATTAGATACCC	Kambhampati^23^
12S	F	12S F1or 12S F2	GATCATTCTAGTTACACCTTCC or GTACAACTACTGTGTTACGACT	N/A
	R	12S reverse	AAACTAGGATTAGATACCC	Kambhampati^23^
16S	F	16S Forward	CGCCTGTTTAACAAAAACAT	Simon *et al*.^24^
	R	16S Reverse	TTTAATCCAACATCGAGG	Cognato *et al*.^25^
16S	F	16S F1	GGAAGGTGTAACTAGAATGATC	N/A
	R	16S R1	GATAGAAACCAACCTGGCTCAC	N/A
COII	F	COII-F	AGAGCWTCACCTATTATAGAAC	Park *et al*.^26^
	R	COII-R	GTARWACRTCTGCTGCTGTTAC	Park *et al*.^26^
COII	F	Modified A-tLeu	CAGATAAGTGCATTGGATTT	Miura *et al*.^27^
	R	B-tLys	GTTTAAGAGACCAGTACTTG	Simon *et al*.^24^
28S	F	Hux	ACACGGACCAAGGAGTCTAAC	Inward *et al*.^7^
	R	Win	GTCCTGCTGTCTTAAGCAACC	Inward *et al*.^7^
H3	F	H3 AF	ATGGCTCGTACCAAGCAGACVGC	Inward *et al*.^7^
	R	H3 AR	ATATCCTTRGGCATRATRGTGAC	Inward *et al*.^7^

N/A: primers were designed for this study.

For PCR amplification, a 25 μL cocktail of 1 μL DNA template, 15.25 μL double-distilled H_2_O (ddH_2_O), 2 μL MgCl_2_ (25 mM), 2.5 μL 10*PCR Loading Buffer, 0.25 μL Taq DNA polymerase (TakaRa DNA kit; 100 mM Tris-HCl, pH8.3, 500 mM KCl), 2 μL dNTP mixture (1 mM concentration of each dNTP) and 1 μL of each primer was used. The PCR conditions included are given in Table S2. The amplified products were electrophoresed in a 1% agarose gel. PCR products were used for sequencing. In the case where sequencing was not successful, purified PCR fragments were cloned and sequenced.

All new sequences were checked for contamination using unrestricted BLAST searches, and NJ trees were produced based on the alignment of each sequenced fragment to check for internal contamination and incorrectly identified GenBank sequences.

### Sequence alignment and phylogenetic analysis

The taxon sample consists of 103 Blattodea taxa (ingroup) and 26 outgroup taxa (Table S3). The molecular data set consists of five genes: the mitochondrial 12S (390 nucleotides, nt), 16S (430nt), COII (730nt), and the nuclear 28S (600nt), H3 (330nt); the total length of the aligned molecular data set is 2831 nt. GenBank sequences were used when available from previous works on Blattodea^7,11–13^, but some problematic sequences were not used in this study, e.g. *Supella longipalpa*. For Mantodea^28^ and others see Table S3. New sequences and their GenBank numbers were listed in Table S3. In our study, names of chimeric taxa (i.e. *Gryllus*, Mantophasmatidae and Oligotomidae) followed Djernæs *et al*.^12^.

Sequences were aligned via the online MAFFT 7 (http://mafft.cbrc.jp/alignment/server/). For ribosomal genes (12S, 16S and 28S), alignments were adjusted according to the first sequence because some ribosomal gene sequences from GenBank were reversed. The Q-INS-i algorithm was selected protein-coding genes (COII, H3), the G-INS-i algorithm was used with other parameters at their default values. Protein-coding genes (COII, H3) were inspected visually and manually corrected in Mega6^29^ after translation into amino acids; few gaps were detected, and alignment was straightforward. Alignments of the ribosomal sequences (12S, 16S and 28S) were inspected visually and manually adjusted in Mega6^29^. Poorly aligned characters were removed but these were limited.

Subsequent analyses were performed on the combined dataset utilizing Maximum likelihood (ML) and Bayesian inference (BI). Bayesian inference (BI) was performed using MrBayes 3.2^30^ and maximum likelihood (ML) was performed using RAxML 7.7.1^31^.

The molecular data set was divided into 9 partitions (partitioned by gene: 12S, 16S, 28S, COII, H3; COII and H3 were divided by codon position (pos1–3)). For ML, the GTRGAMMA model was selected for the combined datasets and 1000 bootstrap replicates were performed. For BI, PartitionFinder v.1.1.1^32^ was used to choose models and model selection was based on BIC. For the 9 partitions, PartitionFinder resulted in the following models: GTR+I+ G: 12S, 16S, COII_pos1, COII_pos2, 28S; TVM+G: COII_pos3; GTR+G: H3_pos1; JC+I: H3_pos2; TVM+I+G: H3_pos3. Two independent sets of Markov chains were run, each with one cold and three heated chains for 1 × 10^7^generations, and every 1000^th^ generation was sampled. Convergence was inferred when a standard deviation of split frequencies <0.01 was completed. Sump and sumt burninfrac were set to 25% and contype was set to allcompat.

### Divergence dating analysis

We performed divergence date analyses based on the combined mitochondrial, nuclear and histone dataset of Blattodea and 26 outgroups (see Table S3). For this analysis, the molecular clock was calibrated using eight minimum age constraints based on termite, cockroach and mantid fossils as shown in Table 2. Analyses were performed using a relaxed molecular-clock model with the Bayesian phylogenetic program BEAST 1.8.0^33^. Rate variation was modeled among branches using uncorrelated lognormal relaxed clocks^33^, with a single model for all genes. A Yule speciation process was used for the tree prior^34^ and posterior distributions of parameters, including the tree, were estimated using MCMC sampling. We performed two replicate MCMC runs, with the tree and parameter values sampled every 5000 steps over a total of 50 million generations. A maximum clade credibility tree was obtained using Tree Annotator within the BEAST software package with a burn-in of 1000 trees. Acceptable sample sizes and convergence to the stationary distribution were checked using Tracer 1.5^33^.

**Table 2:** Fossils Used for Estimation of Divergence Time of Major Clades in the Analysis of Blattodea with 26 outgroup taxa.

Species	Age (Ma)/Minimum Age Constraint for Group	Calibration Group	Soft Maximum Bound (97.5% probability)	Reference
*Baissatermes lapideus*	137	*Cryptocercus* + Isoptera	250	Engel *et al*.^35^
*Baissomantis maculata*	112.6	mantids	250	Grimaldi^36^
*Prochaeradodis enigmaticus*	60	*Hoplocorypha* +S *phodromantis* + *Mantid*	130	Nel & Roy^37^
*Cretaholocompsa montsecana*	125.5	Tiviinae + Holocompsinae + Euthyrrhaphinae + Corydiinae	250	Evangelista*et al*.^38^
*Cratomastotermes wolfschwenningeri*	113	termites	200	Makarkin & Menon^39^
Mastotermitidae indet.	93.5	termites excluding *Mastotermes*	150	Schlüter^40^
*Zootermopsis coloradensis*	33.9	*Zootermopsis* + *Porotermes*	150	James^41^
*Cryptotermes* sp.	16	*Cryptotermes* + *Termes* + *Rhinotermes*	150	Park & Downing^42^

## Results

### Phylogenetic inference

**Figure 1:**
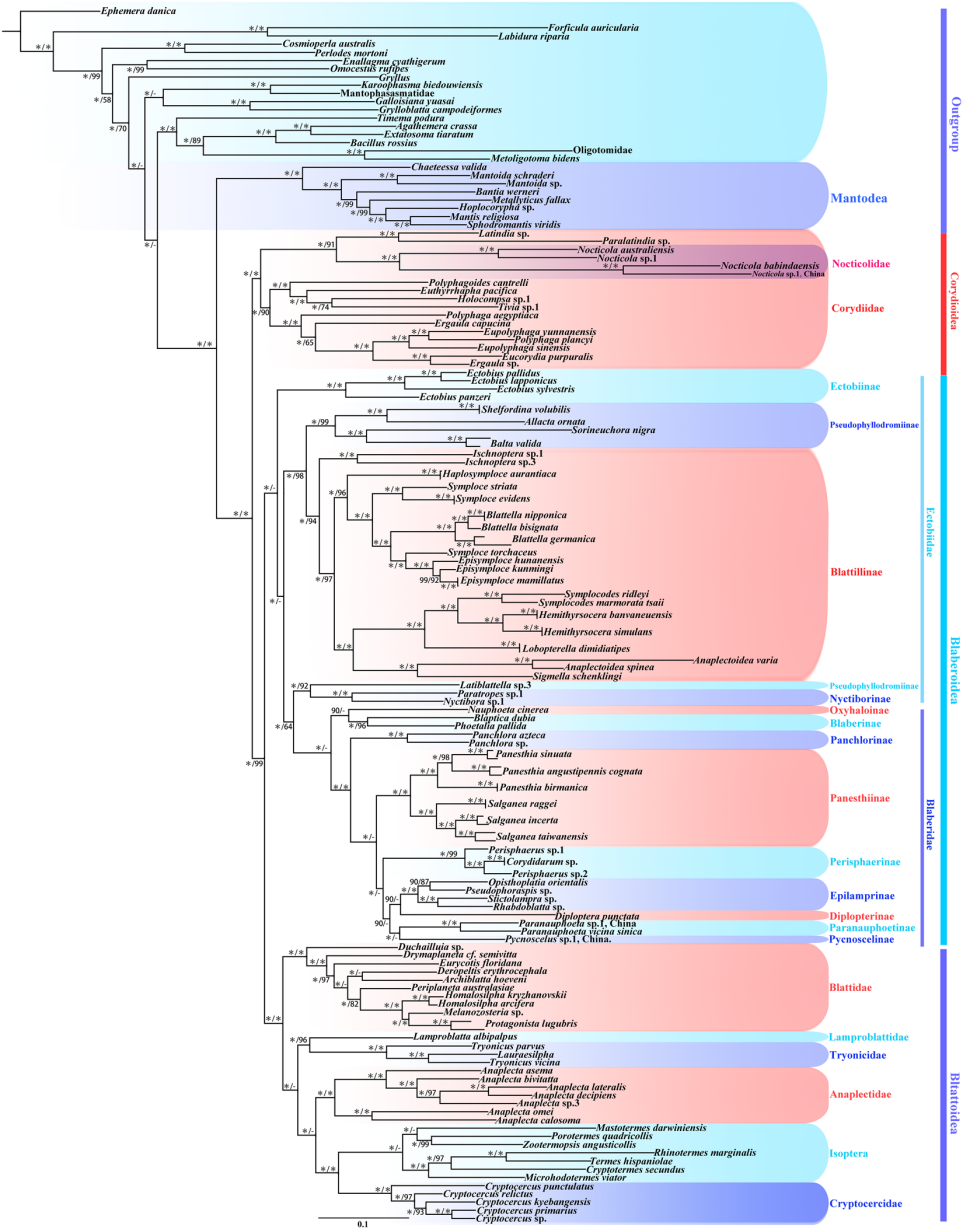
Maximum likelihood (ML) tree derived from analysis of combined data 12S rRNA, 16S rRNA, COII, 28S rRNA and H3 genes. Branch labels are support for our analyses in the following order: bootstrap supports of the maximum-likelihood tree, Bayesian posterior probabilities of the Bayesian tree; dashes (–) indicate that the node is absent for a given analysis; asterisks (*) indicate 100% support for a given analysis. The topology shown was very similar to that derived from BI analysis, with some minor differences (See Fig. S1). Note: Blattillinae = Blattellinae.

For the concatenated dataset (12S rRNA, 16S rRNA, 28S rRNA, COII and H3), phylogenetic analyses yielded essentially identical topologies with generally high support values across the topologies for the two methods utilized (ML and BI) (Figs 1 and S1). Three recognized major lineages of Blattodea from ML and BI inferences were recovered with high support: Corydioidea, Blattoidea and Blaberoidea.

Corydioidea was recovered as sister to the remaining Blattodea (MLB = 100, BPP = 100), and was the first clade within Blattodea. Nocticolidae was recovered as sister group to *Latindia* + *Paralatindia* (MLB = 100, BPP = 100).

Blaberoidea was comprised of Blaberidae and Ectobiidae. In our inferred trees, Ectobiidae was paraphyletic with respect to Blaberidae with high support. All *Ectobius* clustered together and were recovered as the sister to the remaining Blaberoidea (MLB = 100), or to the remaining Ectobiidae (BPP = 46). Nyctiborinae + *Latiblattella* was the sister group of Blaberidae in both methods (BPP = 64, MLB = 100). For four subfamilies of the Blaberidae, (Oxyhaloinae, Blaberinae, Panchlorinae and Panesthiinae) relationships were the same among the two trees; for the remaining subfamilies (Perisphaerinae, Pycnoscelinae, Epilamprinae, Paranauphoetinae and Diplopterinae) there was lower resolution.

Blattidae, Tryonicidae, Lamproblattidae, Anaplectidae, Cryptocercidae and Isoptera formed one large clade, Blattoidea (MLB = 100, BPP = 100). Blattidae was the earliest branching lineage within this clade (MLB = 100, BPP = 100). The topology derived from ML analyses showed that Anaplectidae was the sister group of (Cryptocercidae + Isoptera), followed by Lamproblattidae + Tryonicidae (both MLB = 100). However, in BI analyses, Anaplectidae was recovered to be the sister group of Lamproblattidae + Tryonicidae (BPP = 79), followed by Cryptocercidae + Isoptera (BPP = 99). Cryptocercidae was recovered as the sister group of Isoptera (MLB = 100, BPP = 100). North American *Cryptocercus* species (*Cryptocercus punctulatus*) and Asian species were recovered as sister groups (MLB = 100, BPP = 100).

### Divergence time analysis

The estimated age of the split between Mantodea and Blattodea was 243.6 Ma (204.3 Ma to 289.1 Ma 95% confidence interval [CI]). Corydioidea was recovered as the earliest branching group within Blattodea, having diverged 209.7 Ma (180.5–244.3 Ma 95% CI) from the remaining taxa. The clade comprising Blattidae, Tryonicidae, Lamproblattidae, Anaplectidae, Cryptocercidae and Isoptera diverged from its sister group, Blaberoidea, around 198.3 Ma (173.1–229.1 Ma). The divergence of Blattidae from the remaining group of this clade occurred about 178.2 Ma (155.2–204.4 Ma). The divergence of the lineages leading to termites and *Cryptocercus* was estimated to have occurred 146.4 Ma (137–164.2 Ma 95% CI). American and Asian *Cryptocercus* were estimated to have diverged 67.2 Ma (44.1–96.3 Ma 95% CI). Anaplectidae + (Lamproblattidae + Tryonicidae), was estimated to have arisen 154.8 Ma (133.3–179.3 Ma 95% CI). *Latiblattella* sp.3 from Pseudophyllodromiinae, and *Nyctibora* sp.1 and *Paratropes* sp.1 from Nyctiborinae clustered together, and were recovered as the first clade in Blaberoidea, emerging 183.6 Ma (158.4–214.9 Ma 95% CI) from the remaining Blaberoidea. Blaberidae was found to be monophyletic in this analysis and began to diverge 134.7 Ma (110.6–162.0 Ma) from the remaining Ectobiidae. The lineages leading to most Blattodea species diverged from their sister lineages around 100 Ma or less.

## Discussion

Our analyses using Maximum likelihood (ML) and Bayesian inference (BI) showed that the backbones of the inferred trees were nearly identical, and partly in agreement with previous studies^12^. Three major blattodean lineages, Corydioidea, Blattoidea and Blaberoidea, were recovered with high support values. Our result was markedly different from previous phylogenetic studies based only on morphological characters^6,15,43^. A number of previous molecular studies did not include Anaplectidae^7,8,11,19^, Lamproblattidae^7,8,19^ or Tryonicidae^11^ or combinations of these^7,8^. Legendre *et al*.^13^ included a large number of taxa in their analyses, however several molecular markers were missing for a number of taxa. Ware *et al*.^10^ combined molecular and morphological data of 59 taxa (12 taxa with both molecular and morphological data, and 15 taxa with only morphological data), and used doublet and MK models in MrBayes.

### Placement and monophyly of members within Corydioidea

Corydioidea was found as the sister group to the remaining Blattodea and considered as the basal split within Blattodea with high support (BPP = 100, MLB = 100), consistent with previous studies^7,10^ but not congruent with a recent study^13^. We found Nocticolidae to be monophyletic and firmly nested within Corydiidae with strong support values (BPP = 100, MLB = 100), partially consistent with the results of Djernæs *et al*.^13^ (morphological analyses; molecular and combined analyses). The placement of Nocticolidae found here was not consistent with the proposal that Nocticolidae was the sister group of Corydiidae^7,11^. In Djernæs *et al*.^12^, *N. babindaensis* formed an exceedingly long branch. Similarly, very long branches were found in the *Nocticola* clade in the study of Legendre *et al*.^13^. In our study, *N. babindaensis* (epigean, from Australia) and one Chinese *Nocticola* species (termitophilous, Zhao Tiexiong, pers. obs., from China), were well grouped together and formed two short terminal branches (Figs 1 and S1), also with *N. australiensis* (cavernicolous, from Australia) and *Nocticola* sp. (Cutta Cutta) (cavernicolous, from Australia) as their sister group. The inclusion of our *Nocticola* specimen, the first from outside Australia, provides molecular support for the monophyly of this family. That *N. babindaensis* and *N. australiensis* are placed in different clades is consistent with the notion that *N. babindaensis* and *N. australiensis* are from two different species groups based on the presence or absence of the male tergal gland^44^.

The family Nocticolidae consists of 8 genera, mainly distributed in Madagascar, Australia, Africa and southeastern Asia. It contains representatives with depigmentation and thinning of cuticle, the reduction or loss of eyes, the reduction or loss of tegmina and wings, the elongation and attenuation of appendages, and a more slender body form^45^. Although *Nocticola* representatives show broad morphological similarities to ectobiid cockroaches, the complex and highly variable nature of their genitalia indicates a closer relationship with Corydiidae^44^.

Currently the subfamily Latindiinae is composed of three genera, the type genus *Latindia* with 9 species, *Buboblatta* with 2 species and *Sinolatindia* with 1 species^46,47^. Latindiinae are gracile, delicate, small bodied cockroaches with a number of features similar to ectobiid cockroaches. These include legs weakly covered with spines, long cerci, both sexes winged, and very complex male genitalia^46,48,49^. In both our study and that of Djernæs *et al*.^12^, the placement of *Latindia* + *Paralatindia* as the sister group of Nocticolidae indicates that Latindiinae should be upgraded to the family Latindiidae.

### Placement of Anaplectidae

Consistent with results from a previous molecular study^12^, in our study Anaplectidae had a close genetic relationship with Blattoidea (Blattidae, Tryonicidae, Lamproblattidae, Cryptocercidae and Isoptera) and together formed one large clade, similar to the results of Djernæs *et al*.^12^. Species of *Anaplecta* have a small body size and brown color, and are very similar to ectobiid cockroaches, however they don’t rotate their ootheca before producing them, and the subgenital plate of females is bilobed. Moreover, the male genitalia are more complicated than that of other ectobiids and similar to Blattidae (Fig. S2). It would therefore appear reasonable that *Anaplecta* is closer to Blattoidea than to Ectobiidae.

### Placement and monophyly of Blaberoidea

Within Blaberoidea, Blaberidae is strongly supported to be monophyletic, but Ectobiidae was paraphyletic. This confirmed the results of previous studies^6–13,15^. Compared with Djernæs *et al*.^12^, more ectobiids and blaberids (52 species vs 12 species) were included in our analysis, and our results were quite different. Species of Blaberidae and Ectobiidae each formed monophyletic groups, with the exception of the three ectobiid genera *Nyctibora*, *Paratropes* and *Latiblattella*, which clustered together as the sister group of Blaberidae (BI = 64, MLB = 100). Our finding from ML analysis that Ectobiinae was the earliest branch within the clade Blaberoidea (MLB = 100) is inconsistent with Djernæs *et al*.^11,12^ and Che *et al*.^50^, but, to some extent, similar to the results of Murienne^19^ (Fig. 1). However, in BI analysis, *Ectobius* was recovered as the sister of Pseudophyllodromiinae and Blattellinae, similar to other recent molecular studies^11–13^.

Grandcolas^15^ proposed that Blaberidae was the sister-group of Pseudophyllodromiinae based on morpho-anatomical characters. In contrast, we found Pseudophyllodromiinae (*Latiblattella*) and Nyctiborinae (*Nyctibora* and *Paratropes*) as sister to Blaberidae, similar to Klass^16^. Some Pseudophyllodromiinae representatives (*Supella*, *Balta* and *Margattea* were included) were placed as the sister of Blaberidae^11^, but support values were low.

### Divergence times

**Figure 2:**
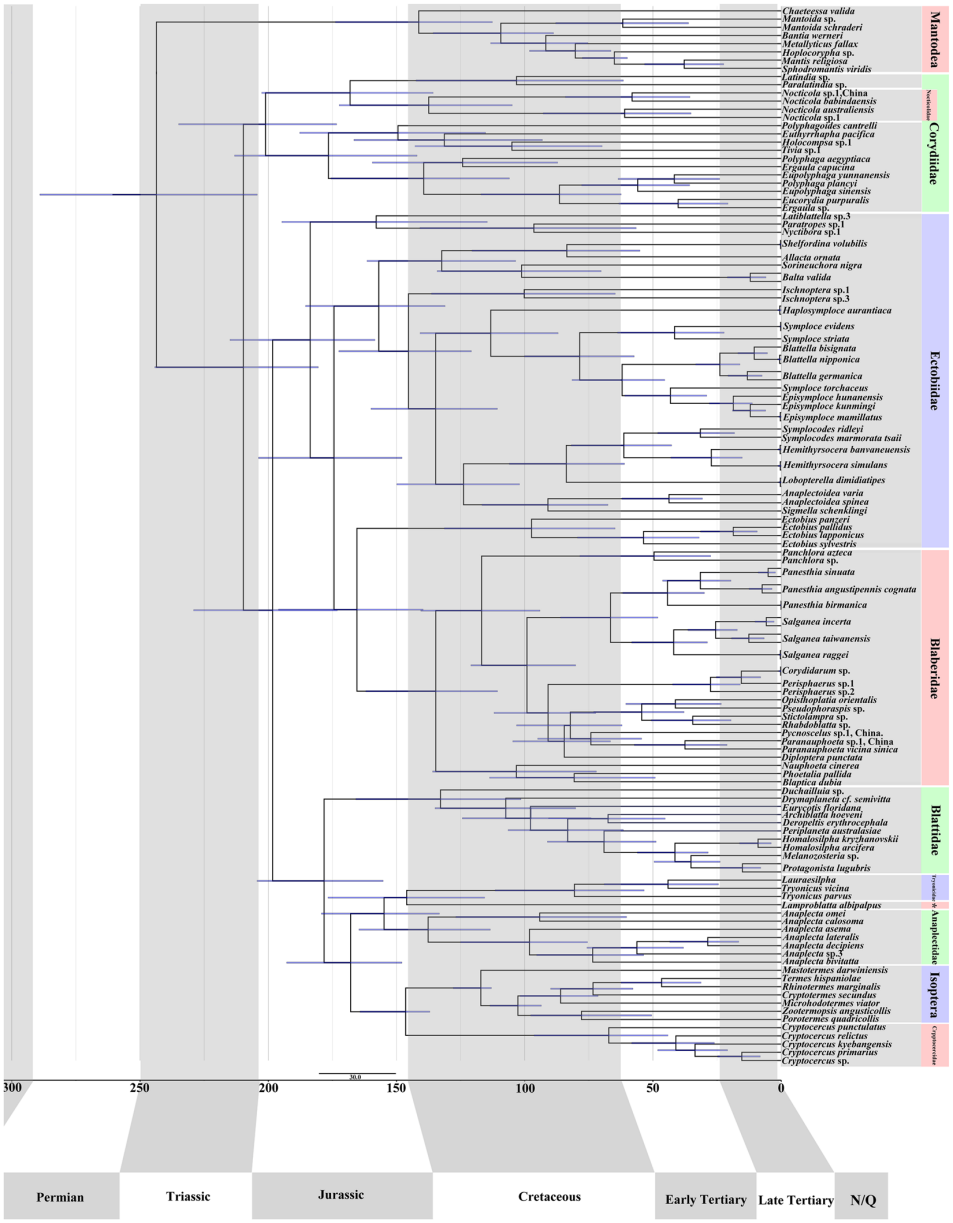
Phylogenetic chronogram of blattodean species based on 12S rRNA, 16S rRNA, COII, 28S rRNA and H3 genes with 26 outgroups, reconstructed using BEAST. Outgroups are not shown. An optimal partitioning scheme was determined by PartitionFinder. Scale bar estimates age in millions of years and blue bars represent 95% highest posterior density intervals for the node ages.

The estimated age of the split between Mantodea and Blattodea (243.6 Ma (204.3–289.1 Ma 95% CI) shown in Fig. 2) is older than some recent estimates around the Triassic-Jurassic boundary (~200 Ma)^51–53^, although much younger than others (Djernæs *et al*.^12^: 273 ± 15 Ma; Legendre *et al*.^13^: ~ 300 Ma). However, the divergence time is much older than that of Che *et al*.^50^ (2017: 155.41 Ma (145.0–185.09 Ma)), which was based on only a single mitochondrial marker.

The divergence of the lineages leading to termites and *Cryptocercus* was estimated to have occurred 146.4 Ma (137–164.2 Ma 95% CI), similar to previous studies (Misof *et al*.^52^: 145 Ma; Tong *et al*.^53^: 140 Ma; Che *et al*.^54^: 145.8 Ma) but younger than others (Djernæs *et al*.^12^: 185 ± 19 Ma; Legendre *et al*.^13^: 195 Ma). The 67.2 Ma (44.1–96.3 Ma 95%CI; Fig. 2) divergence time of the Asian and American *Cryptocercus* lineages is consistent with recent estimates (Che *et al*.^54^: 55.09 Ma (41.55–72.28 Ma); Maekawa *et al*.^18^ 58.7–77.8 Ma). The divergence times of Corydioidea from the remaining Blattodea, and Blattidae from the remaining Blattoidea were estimated beyond 250 Ma and 220 Ma by Djernæs *et al*.^12^, somewhat older than our 209.7 Ma (180.5–244.3 Ma 95% CI) and 178.2 Ma (155.2–204.4 Ma).

Overall our estimated divergence times are younger than those of Djernæs *et al*.^12^ and Legendre *et al*.^13^. One possible reason for this is the selection of fossils for node calibration. In the aforementioned studies, the following were used: 1) a divergence event within Mantodea; 2) the basal split between Mantodea and Blattodea; 3) splits within termites. Calibrating evolutionary rates on the basis of fossils closely related to the taxa under investigation is thought to increase the accuracy of inferred evolutionary timescales^55^.

## Conclusions

This study is a comprehensive analysis of Blattodea phylogeny based on mitochondrial and nuclear genes. Although some deeper nodes are not well resolved, the recovery of major nodal support for the proposed interfamily relationships is an advance over the majority of previous studies. Perhaps the most instructive finding of the present study is the strong effect of additional sampling on Blattodea molecular analyses. For instance, the inclusioin of additional Ectobiidae and Blaberidae representatives appears to greatly influence the resulting Blaberoidea topology. In future reconstructions of cockroach phylogeny, the introduction of samples that better represent the full diversity of the group is therefore recommended.

## Electronic supplementary material

Supplementary Information
