# Erratum: Reconstructing the phylogeny of Blattodea: robust support for interfamilial relationships and major clades

**DOI:** 10.1038/s41598-017-12273-y

**Published:** 2017-09-27

**Authors:** Zongqing Wang, Yan Shi, Zhiwei Qiu, Yanli Che, Nathan Lo

**Affiliations:** 1 College of Plant Protection, Southwest University, Beibei, Chongqing China; 2 School of Life and Environmental Sciences, University of Sydney, Sydney, New South Wales Australia

*Scientific Reports* **7**:3903; doi:10.1038/s41598-017-04243-1; Article published online 20 June 2017

In the Supplementary Information file originally published with this Article, Table S3 was incorrect. This error has been corrected in the Supplementary Information that now accompanies the Article.

