# Supplementary material for: Reconstructing the phylogeny of Blattodea: robust support for interfamilial relationships and major clades
Source: Sci Rep. 2017 Jun 20;7:3903. doi: 10.1038/s41598-017-04243-1 (PMC5478607; doi:10.1038/s41598-017-04243-1)
Supplement: Supplementary file 1 — Supplementary Information [file 41598_2017_4243_MOESM1_ESM.pdf]

Supplementary Data for:

Reconstructing the phylogeny of Blattodea: Robust support for interfamilial relationships and major clades

Zongqing Wang[1], Yan Shi[1], Zhiwei Qiu[1], Yanli Che[1], Nathan Lo[2]*

[1] College of Plant Protection, Southwest University, Beibei, Chongqing, China

[2] School of Life and Environmental Sciences, University of Sydney, Sydney, New South Wales, Australia

* Corresponding author

E-mail: nathan.lo@sydney.edu.au

Table S1. Data for new specimens included in this study

Table S2. PCR conditions used for gene amplification (35 cycles after initial denaturing step)

Table S3. Genbank numbers for included sequences. The colours indicate the sources of the sequences.

Fig S1 Bayesian inference (BI) tree derived from combined data 12S rRNA, 16S rRNA, COII, 28S rRNA and H3 genes. Number above branches representing BPP.

Fig S2. *Anaplecta omei*, A. supra-anal plate (male, ventral view); B. subgenital plate and phallomeres (male, dorsal view); C. supra-anal plate (female, ventral view); D. female genitalia (dorsal view). (All photos are original and taken by the authors).

Table S1. Data for new specimens included in this study

Family	Species	Locality	Collectors
Nocticolidae	*Nocticola* **sp. 1** (China)	Zhuji, Zhejiang	Tiexiong Zhao
Corydiidae	*Eucorydia purpuralis*	Wuyi Mountain, Fujian	Shunhua Gui
	Eupolyphaga sinensis	Xi Mountain, Beijing	Bingqiang Wang
	Eupolyphaga yunnanensis	Weixi, Yunnan	Jianyue Qiu
	Polyphaga plancyi	Zhaocheng, Shandong	Zhong Peng
	Ergaula **sp.**	Wuzhi Mountain, Hainan	Xinran Li, Zhiwei Qiu
Ectobiidae	*Blattella germanica*	Shapingba, Chongqing	Yang Li
	Blattella bisignata	Dahei Mountain, Panzhihua, Sichuan	Keliang Wu
	Blattella nipponica 01	Simian Mountain, Jiangjin, Chongqing	Zongqing Wang
	Blattella nipponica 02	Mojiang, Puhe, Yunnan	Zongqing Wang
	Episymploce hunanensis	Nanling Forest Park, Guangdong	Zaifu Xu
	Episymploce kunmingi	Liang Mountain, Kunming, Yunnan	Dong Wang
	Episymploce mamillatus 01	Zixi Mountain, Chuxiong, Yunnan	Dong Wang
	Haplosymploce aurantiaca 01	Jianfengling Forest Park, Ledong, Hainan	Shunhua Gui, Xinran Li
	Haplosymploce aurantiaca 03	Wuzhishan, Hainan	Shunhua Gui, Xinran Li
	Hemithyrsocera banvaneuensis 01	Dadugang, Jinghong, Yunnan	Xinran Li, Hongguang Liu
	Hemithyrsocera banvaneuensis 02	Dadugang, Jinghong, Yunnan	Xinran Li
	Hemithyrsocera simulans 02	Dadugang, Jinghong, Yunnan	Xinran Li, Hongguang Liu
	Hemithyrsocera simulans 03	Dadugang, Jinghong, Yunnan	Xinran Li, Hongguang Liu
	Sigmella schenklingi 01	Hejiaping, Changyang, Heibei	Xiaojiao Wang
	Symploce evidens 01	Qixianling Forest Park, Baoting, Hainan	Shunhua Gui, Yan Shi
	Symploce ridleyi 03	Menglun, Xishuangbanna, Yunnan	Xinran Li, Hongguang Liu
	Symploce striata 01	Tongmu, Wuyi Mountain, Fujian	Shunhua Gui, Yan Shi
	Symploce torchaceus 01	Diaoluo Mountain, Lingshui, Hainan	Yan Shi
	Lobopterella dimidiatipes 01	Hongshulin Park, Sanya, Hainan	Shunhua Gui
	Anaplectoidea spinea 01	Nabang, Yinjiang, Yunnan	Dong Wang
	Anaplectoidea varia 01	E'mei Mountain, Sichuan	Jinjin Wang
	Allacta ornata 03	Diaoluo Mountain, Lingshui, Hainan	Shunhua Gui, Yan Shi
	Shelfordina volubilis 01	Diaoluo Mountain, Lingshui, Hainan	Shunhua Gui, Yan Shi
	Shelfordina volubilis 02	Diaoluo Mountain, Lingshui, Hainan	Shunhua Gui, Yan Shi
	Sorineuchora nigra 01	Dadugang, Jinghong, Yunnan	Keliang Wu
	Balta valida 01	Dadugang, Jinghong, Yunnan	Xinran Li, Hongguang Liu
	Balta valida 02	Wuzhi Mountain, Hainan	Shunhua Gui, Xinran Li
Blaberidae	*Diploptera punctata* 02	Menglun, Xishuangbanna, Yunnan	Guo Zheng
	Opisthoplatia orientalis 01	Baiwangling, Changjiang, Hainan	Qikun Bai, Lu Qiu
	Paranauphoeta **sp.1**（China）	Limu Mountain, Qiongzhong, Hainan	Xinran Li
	Paranauphoeta vicina sinica 01	Xima Town, Yingjiang, Yunnan	Jianyue Qiu
	Pycnoscelus **sp.1**（China）	Maogan Village, Baoting, Hainan	Xinran Li, Qikun Bai, Lu Qiu

	Panchlora **sp.**	Southwest University, Beibei, Chongqing	Unknown
	Panesthia sinuata 01	Menghai, Yunnan	Jishan Xu, LingXiao Chang
	Panesthia sinuata 02	Menghai, Yunnan	Jishan Xu, LingXiao Chang
	Panesthia angustipennis cognate 01	Beibeng, Motuo, Xizang	Tao Zou
	Panesthia angustipennis cognate 02	Beibeng, Motuo, Xizang	Tao Zou
	Panesthia birmanica 01	Dadugang, Jinghong, Yunnan	Xinran Li
	Panesthia birmanica 02	Dadugang, Jinghong, Yunnan	Xinran Li
	Salganea raggei 01	Wuzhishan, Hainan	Xinran Li
	Salganea raggei 02	Jianfengling Forest Park, Ledong, Hainan	ShunHua Gui
	Salganea incerta 01	Wawushan, Hongya, Sichuan	Yang Li
	Salganea incerta 02	Simianshan, Jiangjin, Chongqing	Hao Xu
	Salganea taiwanensis 01	Daqiutianbao, Jiulianshan Town, Jiangxi	Tao Zou
	Salganea taiwanensis 02	Lianghe Town, Kang County, Gansu	Jinjin Wang
	Perisphaerus **sp.1**	Baigougou, Zunyi, Guizhou	Yuhong Zheng, Xiudan Wang
	Perisphaerus **sp.2**	Diaoluo Mountain, Lingshui, Hainan	Shunhua Gui, Xinran Li
	Corydidarum **sp.**	Bubeng, Mengla, Yunnan	Guo Zheng, Xue Li, Wenyue Zhu
	Corydidarum **sp.**	Bubeng, Mengla, Yunnan	Guo Zheng, Xue Li, Wenyue Zhu
	Pseudophoraspis **sp.**	Limu Mountain, Qiongzhong, Hainan	Xinran Li, Zhiwei Qiu
	Stictolampra **sp.**	Diaoluo Mountain, Lingshui, Hainan	Lu Qiu, Qikun Bai
	Rhabdoblatta **sp.**	Limu Mountain, Qiongzhong, Hainan	Lu Qiu, Qikun Bai
Blattidae s.s.	*Homalosilpha arcifera* 01	Shangyong Town, Mengla, Yunnan	Jianyue Qiu
	Homalosilpha kryzhanovskii 01	Dadugang, Jinghong, Yunnan	Xinran Li
	Protagonista lugubris 01	Longtan Forest Park, Guiping, Guangxi	Shunhua Gui, Xinran Li
	Protagonista lugubris 05	Wangtianshu, Mengla, Yunnan	Jianyue Qiu
	Melanozosteria **sp.**	Limu Mountain, Qiongzhong, Hainan	Xinran Li, Zhiwei Qiu
Anaplectidae	*Anaplecta omei* 01	Campus of Southwest University	Jinjin Wang, Keliang Wu
Cryptocercidae	*Cryptocercus primarius*	Dacaoping, Pingwu, Mianyang, Sichuan	Zongqing Wang, Shunhua Gui
	Cryptocercus **sp.**	Pudacuo, Shangri-la, Diqing, Yunnan	Yan shi, Qikun Bai

Table S2 The PCR conditions used for the genes amplification (35 cycles after initial denaturing)

Genes	Initial denaturing	Denaturation	Annealing	Extension	Finally an extension
12S	95℃/3 min	94℃/30s	49℃/45-60s	72℃/90s	72℃/5 min
16S	94℃/10 min	94℃/45s	48-63℃/45s	72℃/60s	72℃/5 min
28S	94℃/5 min	94℃/30s	55℃/45s	72℃/90s	72℃/7 min
COII	94℃/3 min	94℃/30s	56℃/ 45s	72℃/90s	72℃/7 min
H3	94℃/1 min	94℃/15s	46℃/15s	72℃/15s	72℃/6 min

Table S3. Genbank numbers for included sequences. The colours indicate the sources of the sequences.

Order	Family	Subfamily	Species	12S	28S	COII	H3	16S
**Outgroups**								
Ephemeroptera			*Ephemera danica*	KF855788	KF855809	KF855909	KF855887	KF855860
Dermaptera			*Forficula auricularia*	KF855790	JN615313	KF855911	GU066905	JN615260
			Labidura riparia	*	AY555539/AY7(	AF140544	AY707435	AY144640
Plecoptera			*Cosmioperla australis*	EF623307	EF622852	EF623009	EF622577	EF623148
			Perlodes mortoni	KF855796	KF855814	KF855916	KF855892	KF855870
Odonata			*Enallagma cyathigerum*	KF855787	KF855808	KF855908	KF855886	KF855858
Orthoptera			*Omocestus rufipes*	KF855795	KF855813	KF855915	KF855891	KF855866
			Gryllus	AY560539	AY521799	U88332	AY521719	JX269093
Mantophasmatodea			*Karoophasma biedouwiensis*	KF855791	KF855810	KF855912	KF855889	AY318896
			Mantophasmatidae	DQ874066	DQ874231	DQ874300	GU066922	AY318883
Neoptera			*Galloisiana yuasai*	DQ457209	KC142429	KC142845	DQ457381	JN615261
			Grylloblatta campodeiformes	DQ457227	AY125279	DQ457367	AY125225	DQ457263
Phasmatodea			*Timema podura*	KF855798	KF855816	KF855918	KF855895	KF855873
			Agathemera crassa	Z93282	AY125326	*	AY125269	JN228847
			Extatosoma tiaratum	AB642680	AY125295	FJ474359	FJ474207	Y07552
			Bacillus rossius	GU001956	AY125320	AF148289	AY125263	GU001956
Embioptera			Oligotomidae	AB639034	AY125274	AB639034	AY125221	EU157039
			Metoligotoma bidens	*	EU157052	EU157066	EU157032	EU157040
Mantodea	Chaeteessidae		*Chaeteessa valida* Mn217	FJ806061	*	*	FJ806788	FJ806237
	Mantoididae		*Mantoida schraderi*	EF383155	AY491219	AY491275	AY491333	EF383315
			Mantoida sp. MN110	FJ806015	FJ806576	FJ806904	FJ806740	FJ806190
	Thespidae		*Bantia werneri*	FJ806017	FJ806578	FJ806906	FJ806742	FJ806192
	Metallyticidae		*Metallyticus fallax*	EF383237	EF383721	EF384007	EF384136	EF383397
	Thespidae		*Hoplocorypha* sp. MN190	FJ806050	FJ806616	FJ806941	FJ806777	FJ806226
	Mantidae		*Mantis religiosa*	U17792	FJ806632	EF363226	AY491327	EF383311
			Sphodromantis viridis	FJ805977	FJ806538	GU064720	GU064761	FJ806154
**Ingroups**								
Blattodea	Corydiidae	Latindiinae	*Latindia* sp.	KF855802	KF855822	*	KF855898	*
		Incerta sedis	*Paralatindia* sp.	KF855803	KF855823	*	*	KF855868
	Nocticolidae		*Nocticola australiensis* (Donna Cave)	DQ874070	DQ874234	*	EF203093	KF855864
			Nocticola sp.1 (Cutta Cutta Cave)	KF855794	JN615357	JN615401/EF2(	JN615401/EF	JN615302
			Nocticola babindaensis	KF855785	KF855819	*	KF855879	KF855852
			Nocticola sp. 1 (China)	MF286833	MF286962	*	MF287026	*
	Corydiidae		*Polyphagoides cantrelli*	KF855797	KF855815	KF855917	KF855894	KF855872
		Euthyrrhaphinae	*Euthyrrhapha pacifica*	DQ874050	DQ874218	KF855910	KF855888	KF855861

	Holocompsinae	*Holocompsa* sp. 1	DQ874056	DQ874223	KF855922	*	KF855862
	Tiviinae	*Tivia* sp. 1	DQ874104	DQ874259	KF855919	DQ874021	KF855874
		Polyphaga aegyptiaca	DQ874089	DQ874248	DQ874317	KF855893	KF855871
	Polyphaginae	*Ergaula capucina*	KF855789	DQ874214	DQ874280	DQ873966	JN615300
		Eupolyphaga yunnanensis	MF286819	MF286951	MF287034	MF287031	MF286886
		Polyphaga plancyi	MF286821	MF286953	*	MF287033	MF286895
		Eupolyphaga sinensis	MF286820	MF286952	MF287035	MF287032	MF286887
		Eucorydia purpuralis	MF286822	MF286954	MF287042	*	MF286888
		Ergaula sp.	MF286823	MF286955	MF287075	MF286996	MF286889
Ectobiidae	Ectobiinae	*Ectobius pallidus*	DQ874040	DQ874210	DQ874276	DQ873962	*
		Ectobius lapponicus	DQ874039	DQ874209	DQ874275	DQ873961	*
		Ectobius sylvestris	KF372466	KF372441	*	*	KP986292
		Ectobius panzeri	DQ874041	DQ874211	DQ874277	DQ873963	KF855859
	Pseudophyllodromiinae	*Shelfordina volubilis* 01	MF286777	MF286922	MF287079	MF287023	MF286848
		Shelfordina volubilis 02	MF286778	MF286923	MF287091	MF287024	MF286849
		Allacta ornata 03	MF286776	MF286921	*	MF286969	MF286847
		Sorineuchora nigra 01	MF286783	MF286963	MF287065	MF286992	MF286854
		Balta valida 01	MF286767	MF286898	MF287077	MF286967	MF286836
		Balta valida 02	MF286768	MF286899	MF287078	MF286968	MF286837
Ectobiidae	Blattellinae	*Ischnoptera* sp.1	DQ874059	DQ874226	DQ874293	DQ873979	*
		Ischnoptera sp.3	DQ874024	DQ874194	DQ874261	DQ873944	KF855863
		Haplosymploce aurantiaca 01	MF286779	MF286924	MF287080	MF287014	MF286850
		Haplosymploce aurantiaca 03	MF286780	MF286925	MF287081	MF287015	MF286851
		Symploce striata 01	MF286792	MF286911	MF287064	MF287008	MF286864
		Symploce evidens 01	MF286770	MF286914	MF287068	MF287006	MF286840
		Symploce evidens 02	MF286771	MF286915	MF287069	MF287007	MF286841
		Blattella nipponica 01	MF286789	MF286905	MF287058	MF286994	MF286858
		Blattella nipponica 02	MF286788	MF286910	MF287063	MF286995	MF286863
		Blattella bisignata	MF286787	MF286909	MF287062	MF286997	MF286862
		Blattella germanica	AY536379	DQ874201	DQ874268	DQ873952	EF363265
		Blattella germanica	MF286781	MF286900	MF287085	MF286993	MF286852
		Symploce torchaceus 01	MF286785	MF286903	MF287056	MF287013	MF286856
		Episymploce hunanensis	MF286784	MF286902	MF287055	MF287009	MF286855
		Episymploce kunmingi	MF286786	MF286904	MF287057	MF287010	MF286857
		Episymploce mamillatus 01	MF286790	MF286906	MF287059	MF287011	MF286859
		Episymploce mamillatus 02	MF286791	MF286907	MF287060	MF287012	MF286860
		Symplocodes ridleyi 03	MF286831	MF286916	*	MF287001	MF286842
		Symplocodes marmorata tsaii	MF286769	MF286913	MF287070	MF287000	MF286839

		Hemithyrsocera banvaneuensis 01	MF286774	MF286919	*	MF287004	MF286845
		Hemithyrsocera banvaneuensis 02	MF286775	MF286920	*	MF287005	MF286846
		Hemithyrsocera simulans 02	MF286772	MF286917	*	MF287002	MF286843
		Hemithyrsocera simulans 03	MF286773	MF286918	*	MF287003	MF286844
		Lobopterella dimidiatipes 01	MF286765	MF286896	MF287087	MF286998	MF286834
		Lobopterella dimidiatipes 02	MF286766	MF286897	MF287088	MF286999	MF286835
		Anaplectoidea varia 01	MF286793	MF286964	MF287066	MF287027	MF286865
		Anaplectoidea spinea 01	MF286794	MF286912	MF287067	MF287028	MF286866
		Sigmella schenklingi 01	MF286806	MF286908	MF287061	MF287016	MF286861
	Pseudophyllodromiinae	*Latiblattella* sp. 3	DQ874061	*	DQ874295	DQ873981	*
	Nyctiborinae	*Paratropes* sp. 1	DQ874080	KF855824	DQ874309	DQ873999	KF855869
		Nyctibora sp.1	DQ874072	KF855812	DQ874303	DQ873991	KF855865
Blaberidae	Oxyhaloinae	*Nauphoeta cinerea*	DQ874068	DQ874233	DQ874301	DQ873988	EF363272
	Blaberinae	*Blaptica dubia*	DQ874030	DQ874199	DQ874266	DQ873950	JN615274
		Phoetalia pallida	DQ874086	DQ874245	DQ874314	DQ874004	JN615278
	Panchlorinae	*Panchlora azteca*	DQ874076	DQ874238	DQ874305	DQ873995	KF855867
		Panchlora sp.	*	MF286940	MF287071	MF287022	MF286880
	Panesthiinae	*Panesthia sinuata* 01	MF286797	MF286928	MF287043	MF286972	MF286869
		Panesthia sinuata 02	MF286798	MF286929	MF287044	MF286973	MF286870
		Panesthia angustipennis cognata 01	MF286799	MF286930	MF287045	MF286974	MF286894
		Panesthia angustipennis cognata 02	MF286832	MF286931	MF287048	MF286975	MF286871
		Panesthia birmanica 01	MF286795	MF286926	MF287046	MF286970	MF286867
		Panesthia birmanica 02	MF286796	MF286927	MF287047	MF286971	MF286868
		Salganea raggei 01	MF286804	MF286936	MF287053	MF286980	MF286876
		Salganea raggei 02	MF286805	MF286937	MF287054	MF286981	MF286877
		Salganea incerta 01	MF286802	MF286933	MF287052	MF286978	MF286873
		Salganea incerta 02	MF286803	MF286934	MF287050	MF286979	MF286875
		Salganea taiwanensis 01	MF286800	MF286932	MF287049	MF286976	MF286872
		Salganea taiwanensis 02	MF286801	MF286934	MF287051	MF286977	MF286874
	Perisphaerinae	*Perisphaerus* sp.1	MF286815	MF286947	MF287089	MF286982	*
		Corydidarum sp.	MF286817	MF286949	MF287083	MF286984	*
		Corydidarum sp.	MF286818	MF286950	MF287082	MF286985	*
		Perisphaerus sp.2	MF286816	MF286948	*	MF286983	*
	Epilamprinae	*Opisthoplatia orientalis*	MF286811	MF286943	*	MF286986	MF286883
		Pseudophoraspis sp.	MF286813	MF286945	MF287090	MF286988	*
		Stictolampra sp.	MF286812	MF286944	MF287073	MF286987	MF286884
		Rhabdoblatta sp.	MF286814	MF286946	MF287074	MF286989	MF286885

	Diplopterinae	*Diploptera punctata*	MF286807	MF286938	*	MF286990	MF286878
	Paranauphoetinae	*Paranauphoeta* sp.1, China	MF286810	MF286942	*	MF287030	MF286882
		Paranauphoeta vicina sinica	MF286809	MF286941	MF287072	MF287029	MF286881
	Pycnoscelinae	*Pycnoscelus* sp.1, China	MF286808	MF286939	MF287084	MF286991	MF286879
Blattidae s.s.	Duchailluiinae	*Duchailluia* sp.	KF855786	KF855807	KF855907	KF855885	KF855857
	Polyzostorinae	*Drymaplaneta cf. semivitta*	DQ874038	DQ874208	DQ874274	DQ873960	KF855856
		Eurycotis floridana	DQ874046	JN615351	DQ874283	DQ873968	JN615296
	Blattinae	*Deropeltis erythrocephala*	DQ874035	DQ874205	DQ874271	DQ873957	KF855855
		Archiblatta hoeveni	DQ874026	DQ874196	DQ874262	DQ873946	KF855851
		Periplaneta australasiae	DQ874081	DQ874242	DQ874310	DQ874000	U17808
		Homalosilpha kryzhanovskii 01	MF286825	*	MF287037	MF287020	MF286890
		Homalosilpha arcifera 01	MF286824	MF286956	MF287036	MF287019	*
		Melanozosteria sp.	MF286828	MF286959	MF287039	MF287021	*
		Protagonista lugubris 01	MF286826	MF286957	MF287038	MF287017	MF286838
		Protagonista lugubris 05	MF286827	MF286958	MF287076	MF287018	MF286891
Lamproblattidae		*Lamproblatta albipalpus*	KF855792	KF855821	KF855913	*	JN615295
Tryonicidae		*Tryonicus parvus* (AU)	KF855799	KF855817	KF855920	KF855896	KF855875
		Lauraesilpha	KF855793	KF855811	KF855914	KF855890	EU486045
		Tryonicus vicina (NC)	KF855800	KF855818	KF855921	KF855897	KF855876
Anaplectidae		*Anaplecta asema*	KF855780	KF855804	KF855901	KF855880	*
		Anaplecta bivitatta	KF855781	KF855805	KF855902	KF855881	KF855846
		Anaplecta lateralis	KF855784	KF855806	KF855904	KF855883	KF855849
		Anaplecta decipiens	KF855783	KF855820	KF855899	KF855878	KF855848
		Anaplecta sp. 3	KF855801	*	KF855900	KF855877	KF855850
		Anaplecta omei 01	MF286782	MF286901	MF287086	MF287025	MF286853
		Anaplecta calosoma	KF855782	*	KF855903	KF855882	KF855847
Mastotermitidae	Isoptera	*Mastotermes darwiniensis*	DQ441730	DQ441950	EU253885	GU066915	EF623172
Termopsidae		*Porotermes quadricollis*	DQ441794	DQ442010	DQ442222	*	*
		Zootermopsis angusticollis	DQ441841	AY859614	DQ442267	*	U50778
Rhinotermitidae		*Rhinotermes marginalis*	EU253715	EU253674	EU253890	*	EU253754
Termitidae		*Termes hispaniolae*	EU253733	EU253691	FJ806886	*	FJ806151
Kalotermitidae		*Cryptotermes secundus*	DQ441676	DQ441901	DQ442111	*	KF855854
Hodotermitidae		*Microhodotermes viator*	DQ441739	DQ441959	DQ442169	*	AF262619
Cryptocercidae		*Cryptocercus punctulatus*	DQ441677	DQ441902	AB005462	DQ873955	U38408
		Cryptocercus relictus	AY631411	*	KF855906	*	AB078597
		Cryptocercus kyebangensis	AF310221	FJ806522	KF855905	KF855884	KF855853
		Cryptocercus primarius	MF286830	MF286960	MF287041	MF286966	MF286893
		Cryptocercus sp.	MF286829	MF286961	MF287040	MF286965	MF286892

Main sequence sources (colour coded):

Sequenced for present study
Djernæs et al. (2015)
Inward et al. (2007a)
Svenson and Whiting (2009)
Djernæs et al. (2012)
Whiting et al. (2003)
Svenson and Whiting (Unpubl.)
Inward et al (2007b)
Legendre et al. (2008)
Legendre et al. (2014)
Legendre et al. (2015)
Terry and Whiting (Unpubl.)
Svenson and Whiting (2004)
Jarvis and Whiting (2006)
Various others

References:

Djernæs, M., Klass, K.-D., Picker, M.P. & Damgaard, J. (2012). Phylogeny of cockroaches (Insecta, Dictyoptera, Blattodea), with placement of aberrant taxa and exploration of out-group sampling. Systematic Entomology **37:** 65-83.

Djernæs, M., Klass, K.-D. & Eggleton, P. (2015). Identifying possible sister groups of Cryptocercidae+Isoptera: A combined molecular and morphological phylogeny of Dictyoptera. Molecular Phylogenetics and Evolution **84**: 284-303.

Inward, D., Beccaloni, G. & Eggleton, P. (2007a). Death of an order: a comprehensive molecular phylogenetic study confirms that termites are eusocial cockroaches. Biological Letters **3**: 331-335.

Inward, D., Vogler, A.P. & Eggleton, P. (2007b). A comprehensive phylogenetic analysis of termites (Isoptera) illuminates key aspects of their evolutionary biology. Molecular Phylogenetics and Evolution **44**: 953–967.

Legendre, F., Whiting, M.F., Bordereau, C., Cancello, E.M., Evans, T.A. & Grandcolas, P. (2008) The phylogeny of termites (Dictyoptera: Isoptera) based on mitochondrial and nuclear markers: implications for the evolution of the worker and pseudergate castes, and foraging behaviors. Molecular Phylogenetics and Evolution **48**: 615–627.

Jarvis, K.J. & Whiting, M.F. (2006) Phylogeny and biogeography of ice crawlers (Insecta: Grylloblattodea) based on six molecular loci: designating conservation status for Grylloblattodea species. Molecular Phylogenetics and Evolution **41**: 222–237.

Svenson, G.J. & Whiting, M.F. (2004) Phylogeny of Mantodea based on molecular data: evolution of a charismatic predator. Systematic Entomology **29**: 359–370.

Svenson, G.J., Whiting, M.F. (2009). Reconstructing the origins of praying mantises (Dictyoptera, Mantodea): the roles of Gondwanan vicariance and morphological convergence. Cladistics **25**: 468-514.

Whiting, M.F., Bradler, S. & Maxwell, T. (2003) Loss and recovery of wings in stick insects. Nature **421**, 264–267.

Legendre,F., D'Haese,C.A., Deleporte,P., Pellens,R., Whiting,M.F., Schliep,K. & Grandcolas,P.(2014) The evolution of social behaviour in Blaberid cockroaches with diverse habitats and social systems: phylogenetic analysis of behavioural sequences. Biological Journal of the Linnean Society **111**: 58-77.

Legendre,F., Nel,A., Svenson,G.J., Robillard,T., Pellens,R.& Grandcolas,P. (2015) Phylogeny of Dictyoptera: Dating the Origin of Cockroaches, PrayingMantises and Termites with Molecular Data and Controlled Fossil Evidence. PLoS ONE **10**: 1-27.

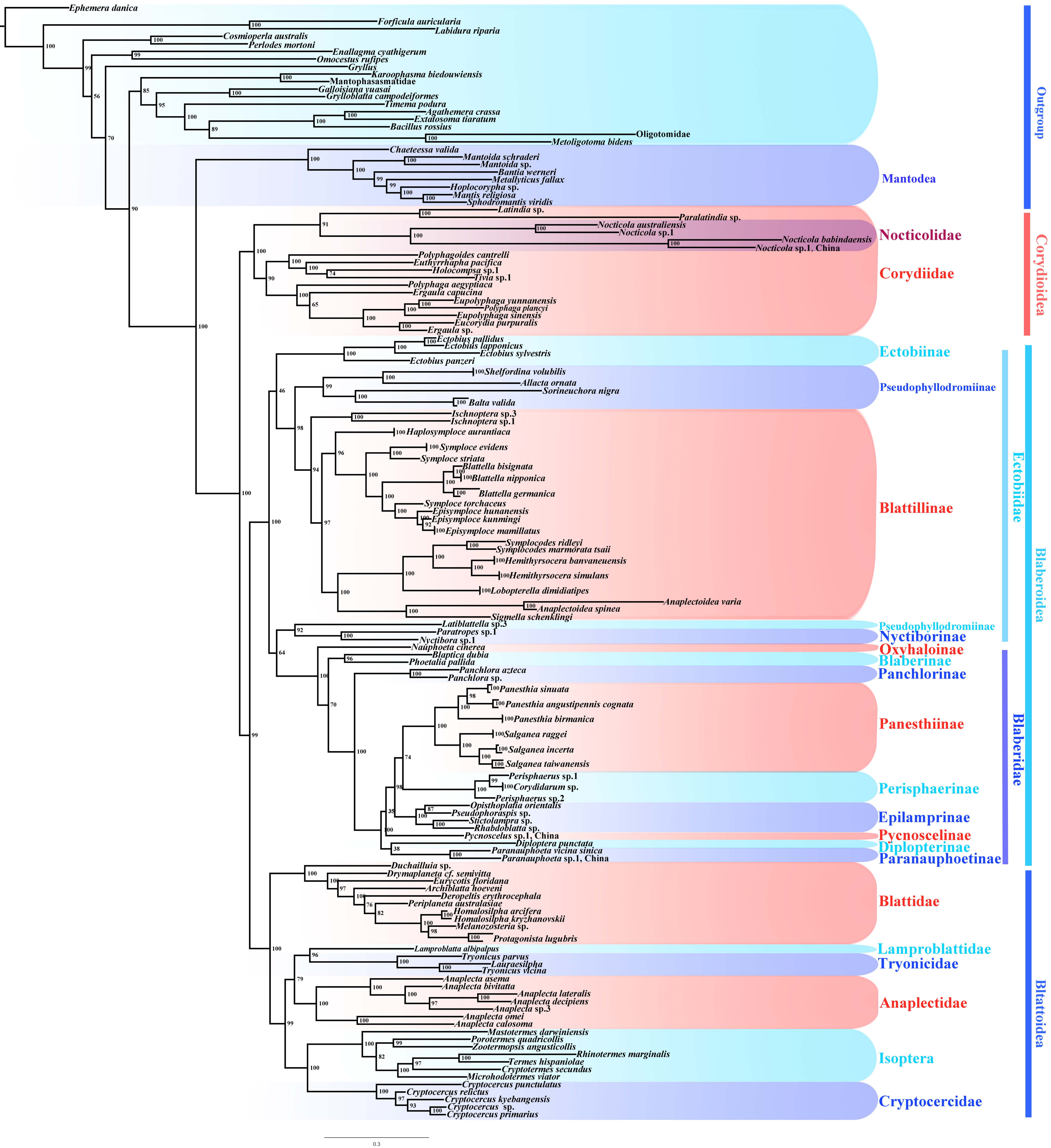

Ephemera danica
Forficula auricularia
Labidura riparia
Cosmioperla australis
Perlodes mortoni
Enallagma cyathigerum
Omocestus rufipes
Gryllus
Karoophasma biedouwiensis
Mantophasmatidae
Galloisiana yuasai
Grylloblatta campodeiformes
Timema podura
Agathemera crassa
Extatosoma tiaratum
Bacillus rossius
Oligotomidae
Metoligotoma bidens
Outgroup
Chaeteessa valida
Mantoida schraderi
Mantoida sp.
Bantia werneri
Metallyticus fallax
Hoplocorypha sp.
Mantis religiosa
Sphodromantis viridis
Mantodea
Latindia sp.
Paralatindia sp.
Nocticola australiensis
Nocticola sp.1
Nocticola babindaensis
Nocticola sp.1, China
Nocticolidae
Polyphagoides cantrelli
Euthyrrhapha pacifica
Holocompsa sp.1
Tivia sp.1
Polyphaga aegyptiaca
Ergaula capucina
Eupolyphaga yunnanensis
Polyphaga plancyi
Eupolyphaga sinensis
Eucorydia purpuralis
Ergaula sp.
Corydiidae
Corydioidea
Ectobius pallidus
Ectobius lapponicus
Ectobius sylvestris
Ectobius panzeri
Ectobiinae
Shelfordina volubilis
Allacta ornata
Sorineuchora nigra
Balta valida
Pseudophyllodromiinae
Ischnoptera sp.3
Ischnoptera sp.1
Haplosymploce aurantiaca
Symploce evidens
Symploce striata
Blattella bisignata
Blattella nipponica
Blattella germanica
Symploce torchaceus
Episymploce hunanensis
Episymploce kunmingi
Episymploce mamillatus
Symplocodes ridleyi
Symplocodes marmorata tsaii
Hemithyrsocera banvaneuensis
Hemithyrsocera simulans
Lobopterella dimidiatipes
Anaplectoidea varia
Anaplectoidea spinea
Sigmella schenklingi
Blattillinae
Latiblattella sp.3
Paratropes sp.1
Nyctibora sp.1
Pseudophyllodromiinae
Nyctiborinae
Ectobiidae
Nauphoeta cinerea
Blaptica dubia
Phoetalia pallida
Panchlora azteca
Panchlora sp.
Oxyhaloinae
Blaberinae
Panchlorinae
Panesthia sinuata
Panesthia angustipennis cognata
Panesthia birmanica
Salganea raggei
Salganea incerta
Salganea taiwanensis
Panesthiinae
Perisphaerus sp.1
Corydidarum sp.
Perisphaerus sp.2
Perisphaerinae
Opisthoplatia orientalis
Pseudophoraspis sp.
Stictolampra sp.
Rhabdoblatta sp.
Epilamprinae
Pycnoscelus sp.1, China
Pycnoscelinae
Diploptera punctata
Diplopterinae
Paranauphoeta vicina sinica
Paranauphoeta sp.1, China
Paranauphoetinae
Blaberidae
Blaberoidea
Duchailluia sp.
Drymaplaneta cf. semivitta
Eurycotis floridana
Archiblatta hoeveni
Deropeltis erythrocephala
Periplaneta australasiae
Homalosilpha arcifera
Homalosilpha kryzhanovskii
Melanozosteria sp.
Protagonista lugubris
Blattidae
Lamproblatta albipalpus
Lamproblattidae
Tryonicus parvus
Lauraesilpha
Tryonicus vicina
Tryonicidae
Anaplecta asema
Anaplecta bivitatta
Anaplecta lateralis
Anaplecta decipiens
Anaplecta sp.3
Anaplecta omei
Anaplecta calosoma
Anaplectidae
Mastotermes darwiniensis
Porotermes quadricollis
Zootermopsis angusticollis
Rhinotermes marginalis
Termes hispaniolae
Cryptotermes secundus
Microhodotermes viator
Isoptera
Cryptocercus punctulatus
Cryptocercus relictus
Cryptocercus kyebangensis
Cryptocercus sp.
Cryptocercus primarius
Cryptocercidae
Bltattoidea
0.3

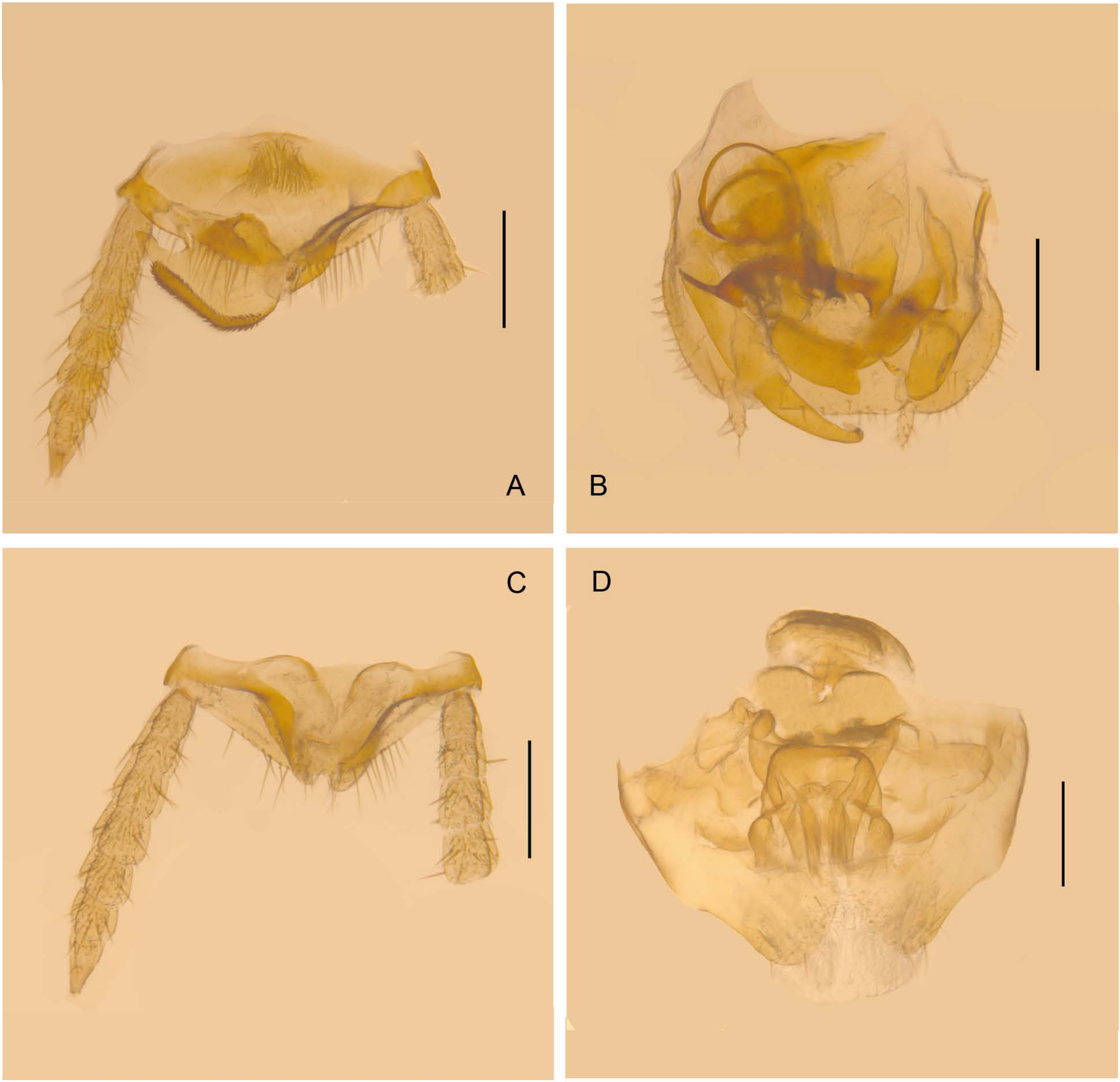
A
B
C
D
